# Repression of Akt3 gene transcription by the tumor suppressor RIZ1

**DOI:** 10.1038/s41598-018-19943-5

**Published:** 2018-01-24

**Authors:** Qingnan Liu, Xiaotian Qu, Xiaolei Xie, Pei He, Shi Huang

**Affiliations:** 1 Center for Medical Genetics, School of Life Sciences, Central South University, Changsha, Hunan China; 2 Department of Pathology, YiYang Medical College, Yiyang, Hunan China

## Abstract

RIZ1 has been studied as a tumor suppressor and may play a role in metabolic diseases related to the Western style diet, such as cancer and obesity. The Akt pathway is known to play a role in both cancer and obesity, and a link between Akt and RIZ1 has also been found. To better understand the role of RIZ1 in obesity and cancer, we investigated how RIZ1 regulates the expression of Akt3. We found that overexpression of RIZ1 in HEK293 cells reduced the expression of Akt3 protein. Luciferase reporter activity of Akt3 gene promoter was significantly reduced in cells co-transfected with RIZ1. Recombinant proteins of RIZ1 was able to bind the Akt3 promoter *in vitro*, and chromatin immunoprecipitation assay also demonstrated the ability of RIZ1 binding to the Akt3 promoter *in vivo*. Overexpression of RIZ1 increased H3K9 methylation on the Akt3 promoter. These results identify Akt3 as a target of RIZ1 regulation and expand our understanding of the Akt pathway in cancer and obesity.

## Introduction

RIZ1/PRDM2/KMT8, belonging to the PR (PRDI-BF1 and RIZ1 homology) domain containing histone lysine methyltransferase family, was first isolated in a functional screening for retinoblastoma-binding protein^1,2^. It was one of the earliest recognized genes that commonly undergo DNA methylation-mediated gene silencing in numerous human tumor types^3–5^. And among the two different length products of PRDM2, RIZ1 and RIZ2, it is only the longer protein RIZ1 that is frequently silenced in tumors^6,7^. Mouse gene knockout models also show that RIZ1 inactivation (but not RIZ2) can lead to tumor susceptibility^8^. Frameshift mutations in RIZ1, affecting (A)8 or (A)9 repeats within exon 8, are common in tumors with microsatellite instability^9–11^. In an unbiased scan for genes with frameshift mutations, RIZ1 was identified as one of the few commonly mutated genes in stomach cancer (while most genes with microsatellites stay unaffected), leading to the conclusion that RIZ1 is a cancer driver gene^12^. Consistently, correcting RIZ1 frameshift mutations in colorectal cancer cells increased global histone 3 lysine 9 (H3K9) dimethylation and reduced tumor growth^13^.

The zinc finger motifs of RIZ1 are important for transcriptional repression, protein interactions^14,15^, and DNA binding^16,17^. RIZ1 has been found to recognize GGGCGG, E-box (CANNTG), and CTCATATGAC type elements. The PR domain is closely related to the SET domain and is responsible for RIZ1 methyltransferase activity on H3K9^18,19^.

Cancer and obesity share common environmental risk factors and biological pathways. The phosphatidylinositol 3-kinase (PI3K)/V-Akt murine thymoma viral oncogene homolog (Akt) (PKB)/mechanistic target of rapamycin (mTOR) cascade is a key signaling pathway linking obesity and cancer and regulates cell proliferation, apoptosis, and metabolism^20,21^. Akt is commonly activated by phosphorylation in cancers^22^. The Akt pathway involves three distinct isoforms, Akt1, Akt2, and Akt3. Akt1 is ubiquitously expressed, Akt2 is highly expressed in insulin-responsive tissues such as adipose tissue, liver and skeletal muscle, and Akt3 is highly expressed in brain^23,24^. They may regulate cell proliferation, motility and invasion^25^.

A role of RIZ1 in insulin-like growth factor (IGF1) and Akt signaling has been reported^26^. Our previous studies found that RIZ1 knockout (KO) mouse gained more weight on a high fat diet. They had higher Akt3 mRNA levels and activated PI3K/Akt/mTOR pathway^27^. We here studied the regulation of Akt3 gene promoter by RIZ1. We found that RIZ1 binds to the promoter region of Akt3 and represses its expression, which was associated with methylation of H3K9.

## Results

### RIZ1 on Akt3 expression in tissue culture

**Figure 1:**
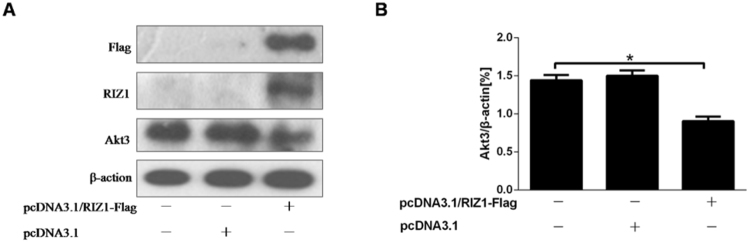
RIZ1 overexpression on Akt3 protein levels. (**A**) The western blotting analysis for Flag, RIZ1, Akt3 and β-actin. (**B**) Results from densitometric analysis of Akt3 protein level relative to β-actin from 3 independent experiments was shown as means ± SD; *p < 0.05.

Previous studies by our group have shown that RIZ1 could regulate the activity of Akt and influence the mRNA expression of Akt3 in mouse^27^. Here, we further assessed whether the expression of Akt3 could be regulated by RIZ1. As shown in Fig. 1, overexpression of RIZ1 was accompanied by a significant decline in Akt3 protein level.

**Figure 2:**
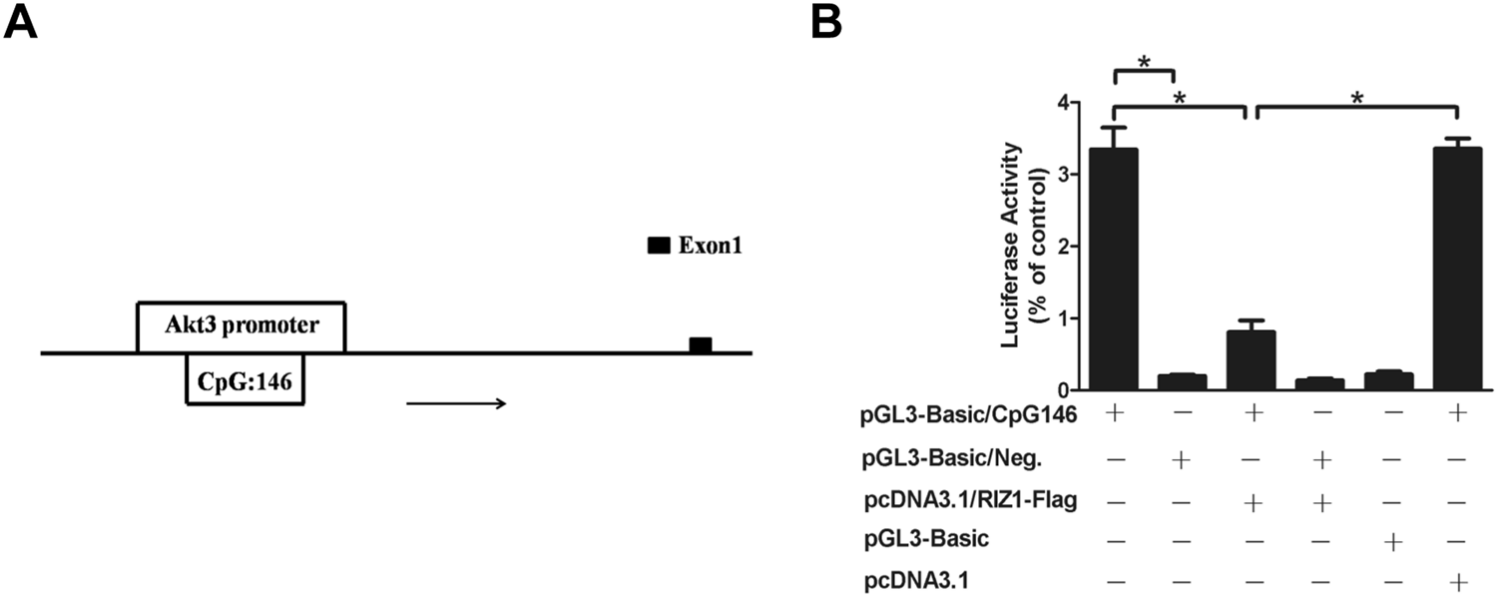
RIZ1 on Akt3 gene promoter activity. (**A**) Schematic diagram of mouse Akt3 gene promoter were found from http://www.ensembl.org, which included the CpG island 146 (Data were indentified from University of California Santa Cruz (UCSC)). (**B**) The Akt3 promoter plasmid pGL3-Basic/CpG146 and the control plasmid pGL3-Basic/Neg. (negative control) was co-transfected with RIZ1-Flag expression plasmid into HEK293 cells. Dual luciferase activity was measured by a luminometer after 48 h transfection. The values represent means ± SD; n = 3; *p < 0.05.

RIZ1 has been shown previously by reporter assays to regulate transcription^17^, so we next performed luciferase reporter assay to test RIZ1 effect on Akt3 promoter. We identified and cloned a candidate promoter containing a 1785 bp long GC rich fragment (including CpG island #146 as indexed by Ensemble database) located at the 5′-region of the mouse Akt3 gene (Fig. 2A). And we also cloned a 718 bp long fragment from Akt3 gene 3′-region as a negative control. The results showed that the 5′-fragment but not the 3′-fragment was able to support the expression of the reporter gene in pGL3-Basic vector (Fig. 2B). Co-transfection of RIZ1-Flag plasmid and the Akt3 promoter reporter plasmid pGL3-Basic/CpG146 led to decreased luciferase activity (Fig. 2B).

### RIZ1 binding to the predicted sites of Akt3 gene promoter

**Figure 3:**
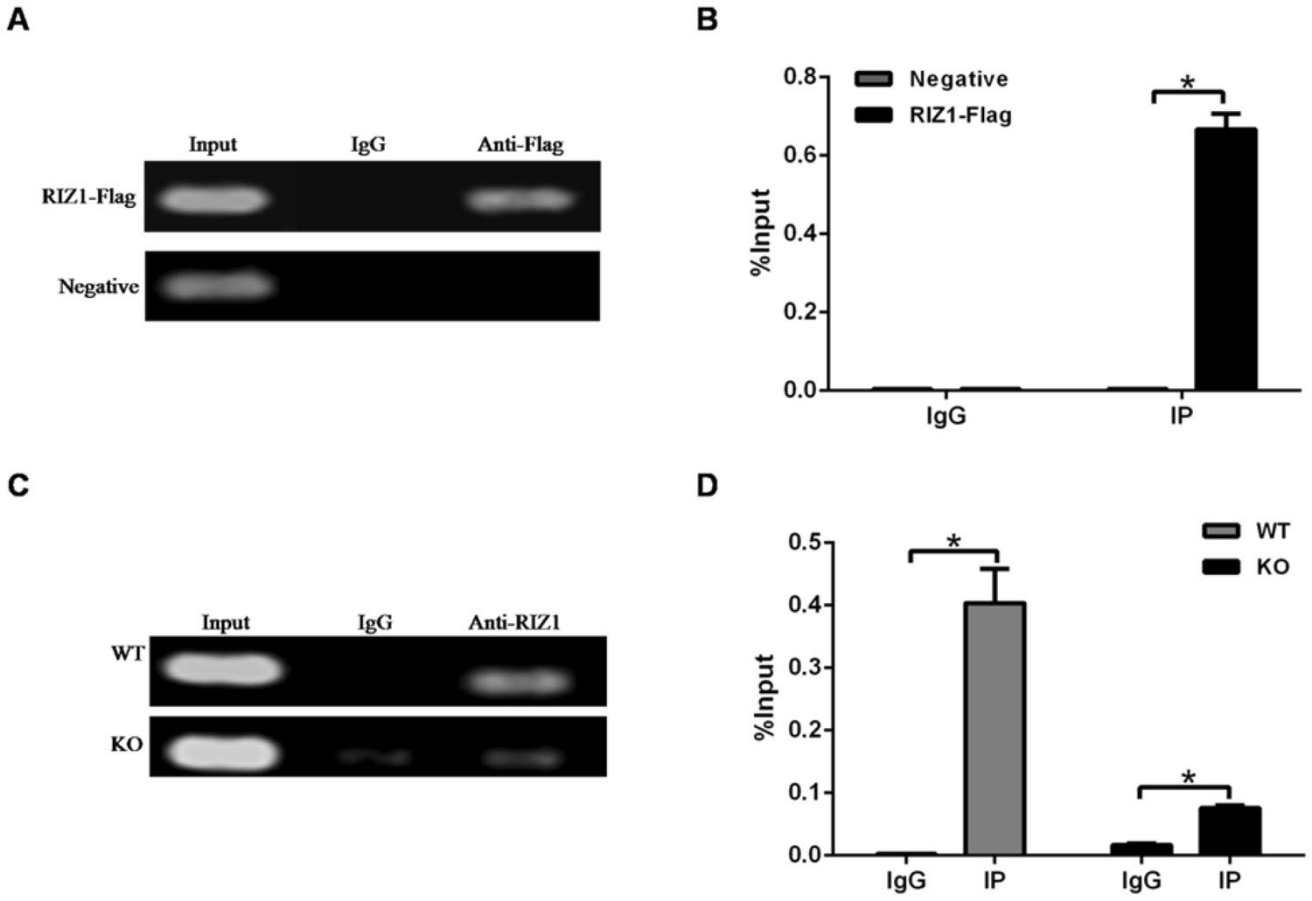
Akt3 gene promoter ChIP assay. (**A**) ChIP in transfected cells. HEK293 cells were transfected with pcDNA3.1/RIZ1-Flag or pcDNA3.1 vectors (Negative). PCR was performed using primers encompassing human Akt3 promoter region CpG 166 (chr1:243849411-243851290). (**B**) ChIP-qPCR assay in transfected cells. Data represent mean ± SD of triplicate measurements. *p < 0.05. (**C**) ChIP in mouse tissues. Liver tissues from 5 weeks old RIZ1 WT and KO mice were used for ChIP assay. PCR was performed using primers encompassing mouse Akt3 promoter region CpG 146 (chr1:177257300-177259121). (**D**) ChIP-qPCR assay in mouse livers. Data represent mean ± SD of triplicate measurements. *p < 0.05.

Since the above results indicated RIZ1 regulation of Akt3 promoter, we next determined whether RIZ1 could directly bind to Akt3 promoter. Based on mouse and human genomic sequences of Akt3 gene available from the Ensembl database, we identified two CpG rich sites as candidate RIZ1 binding sites, including CpG:166 in the human gene and CpG:146 in the mouse gene (numbers mark the CpG counts as indexed by Ensemble). We designed PCR primers to cover these sites and performed chromatin immunoprecipitation (ChIP) assays to detect RIZ1 binding to these sites. RIZ1 binding was found in RIZ1-Flag plasmid transfected cells (Fig. 3A and B) and in wild type (WT) mouse liver (Fig. 3C and D).

**Figure 4:**
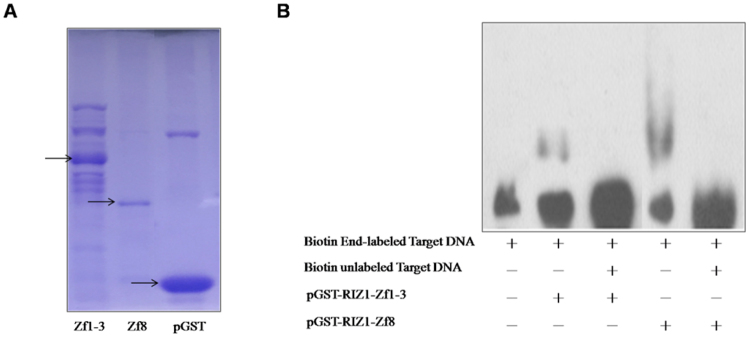
Binding to Akt3 promoter by recombinant RIZ1 protein fragments. (**A**) Shown is a Coomassie Blue stained gel of partially purified GST fusion proteins of RIZ1 containing zinc finger motifs 1–3 and 8 (pGST-RIZ1-Zf1–3 and Zf8). The arrow from left to right pointed to the protein bands of Zf1-3, Zf8 and pGST respectively. (**B**) EMSA results. EMSA was performed using purified pGST-RIZ1-Zf1-3 and Zf8 fusion proteins and biotin end labeled probes of Akt3 promoter region CpG146. Un-labeled probes (30 fold excess) were used in competition experiments.

To further verify RIZ1 binding to the Akt3 promoter, we performed electrophoresis mobility shift assay (EMSA) using recombinant GST-fusion proteins containing RIZ1 zinc finger motifs 1 to 3 (Zf1-3) and 8 (Zf8) (Fig. 4). Binding to the mouse promoter fragment CpG:146 was found for the RIZ1 Zf1-3 and the Zf8 proteins, which was abolished by an excess of unlabeled probe (Fig. 4B). However, these short truncated forms of RIZ1 could not affect Akt3 promoter reporter in co-transfection assays (data not shown).

### Regulation of H3K9 methylation of Akt3 promoter by RIZ1

**Figure 5:**
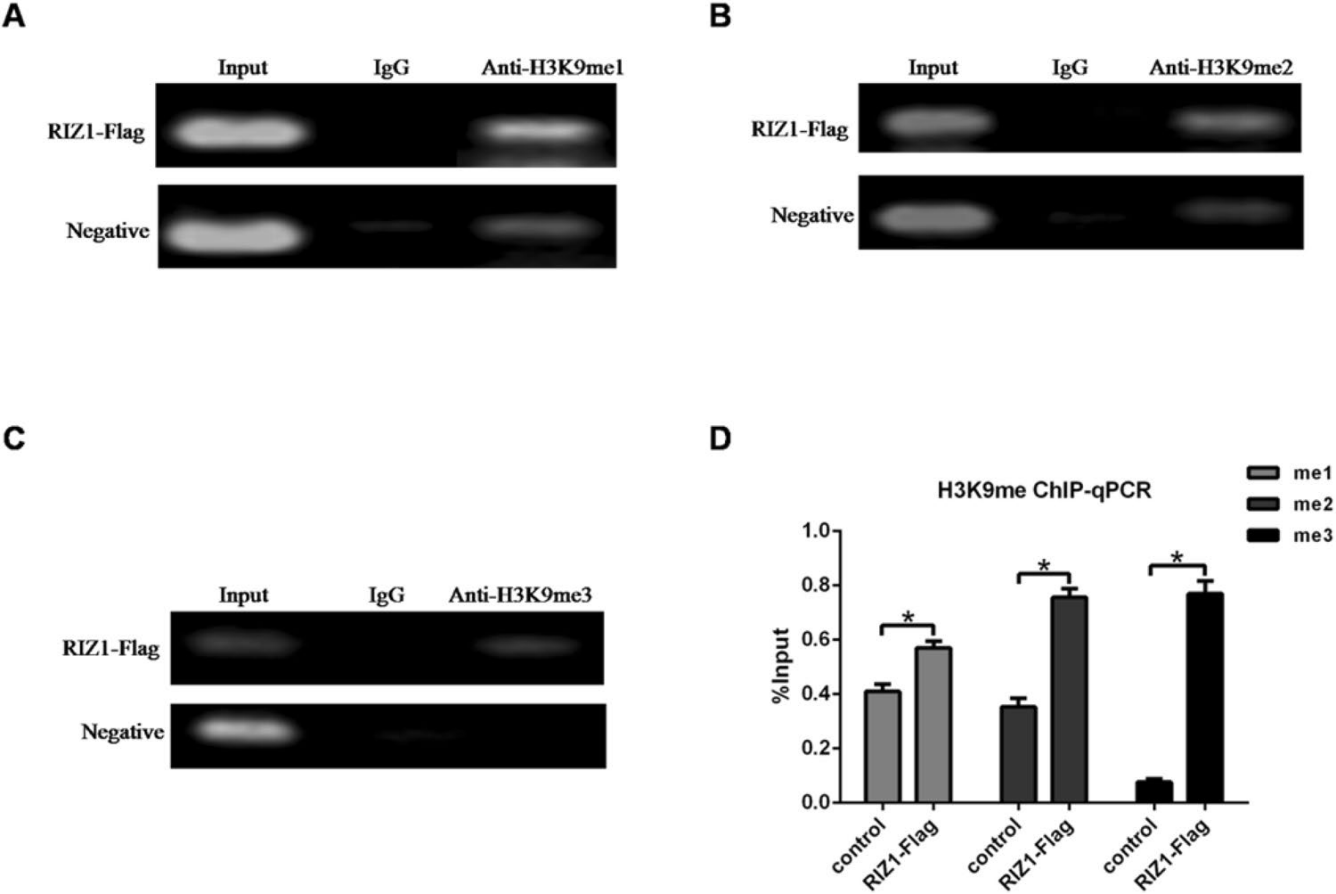
RIZ1 effect on H3K9 methylation on Akt3 promoter. ChIP assays using anti-H3K9me1 (**A**), anti-H3K9me2 (**B**), anti-H3K9me3 (**C**) antibodies were performed with HEK293 cells overexpressing Flag-tagged RIZ1. ChIP-qPCR assay was also performed (**D**). Data represent mean ± SD of triplicate measurements. *p < 0.05.

RIZ1 was a member of the SET/PR domain histone lysine methyltransferase family. We next examined RIZ1 effect on H3K9 methylation of Akt3 promoter. We performed ChIP-qPCR analysis with antibodies against transcriptionally repressive histone marks including H3K9me1, H3K9me2, and H3K9me3 (Fig. 5). In RIZ1-Flag transfected cells, we found increased levels of H3K9 mono-, di-, and tri-methylation in the Akt3 promoter.

## Discussion

Estrogen receptor-negative breast cancer and androgen-independent prostate cancer lines show high levels of Akt3 mRNA^28^. In Akt3-knockdown cells, mitochondrial oxygen consumption rate was significantly reduced, indicating a critical role of Akt3 in mitochondrial respiration in human cancer cells^29^. And importantly, enhanced PI3K/Akt3 pathway activity is one of the main contributors in the genesis of melanoma^30^. Deregulated Akt activity has been shown to be responsible for 35–70% of advanced metastatic melanomas, and the majority of melanomas have elevated Akt3 expression and activity^31^. Akt3 may play an important role in the development of the disease and drug resistance^32,33^. Targeted inhibition of Akt3 in melanoma cell lines could decrease the pAkt level significantly^30,34^. However, the mechanism leading to deregulation of Akt3 remains to be better understood^35^. We in this study identified RIZ1 as a candidate repressor of Akt3 transcription. Our findings were consistent with known roles of RIZ1 and Akt3 in cancer pathogenesis. Future studies will be needed to determine whether RIZ1 (but not RIZ2) deregulation and Akt3 mis-expression may be coupled.

Insulin and Akt signaling modulates adipose tissue growth and adipogenesis^36^. Mouse embryonic fibroblasts (MEFs) lacking Akt display an inability to differentiate into adipocytes^37^, an RNAi-mediated decrease in Akt was found to block the differentiation of 3T3-L1 cells^38^. Since RIZ1 KO mouse exhibit diet-induced obesity and higher body weight, the link between RIZ1 and Akt3 found here may also play a role in obesity related pathways.

We here found that RIZ1 could bind to the promoter of Akt3 and suppress its expression. Like its related PR domain genes, RIZ1 also displays properties of transcription factors with a potential role in cell growth and tumorigenesis. RIZ1 gene products encode DNA-binding as well as transcription factor-binding activities^1,16,39^.

Recent studies provided evidence that malignant transformation results from a complex interplay of both genetic and epigenetic alterations affecting cell cycle regulation, cell proliferation, apoptosis, invasion, and angiogenesis, which finally lead to malignant phenotype development^40–42^. Histone methylation has an important role in transcriptional regulation^43^. Among them, H3K9 can be mono-, di-, or trimethylated and then induces distinct effects on chromatin compaction and gene expression^44^. The results here showing RIZ1 regulation of histone methylation on Akt3 promoter may help elucidate the link between histone methylation and cancer/obesity.

## Materials and Methods

### Cell culture

HEK293 cell line, purchased from the American Type Culture Collection-ATCC (Manassas, VA), were cultured in Dulbecco’s modified Eagle medium (DMEM) supplemented with 10% (v/v) fetal bovine serum (FBS, GE Healthcare Life Sciences), 4 mM L-glutamine, 4500 mg/l glucose at 37 °C under an atmosphere of 5% CO_2_.

### Animals

The present study was reviewed and approved by the State Key Laboratory of Medical Genetics animal center of Central South University (licensing ID KYXK(Hunan)2015-0014), and it conformed to the guiding principles of the ‘Guide for the Care and Use of Laboratory Animals.’ RIZ1 KO mice in 129 Sv/C57BL6 background were crossed to 129 Sv mice to produce RIZ1 KO mice in 129 Sv background^45^. Mice were maintained on a 12:12 hour light/dark cycle given food ad libitum. Five weeks old male animals of WT and KO genotype were used to do experiments.

### Sodium dodecyl sulfate-polyacrylamide gel electrophoresis (SDS-PAGE) and immunoblotting

Cell lysates were prepared by incubating for 30 min in a buffer containing 25 mM Tris (pH 7.5), 75 mM NaCl, 5% glycerol, 2% SDS and protease/phosphatase inhibitors (Sigma) followed by centrifuging at 10,000 g for 10 min at room temperature. The protein concentration was measured using the BCA protein assay kit (Thermo Scientific). Proteins were separated by SDS-PAGE and transferred electrophoretically onto polyvinylidene fluoride (PVDF) membranes (Millipore), which were incubated overnight with the primary antibodies including mouse monoclonal anti-Akt3 (Santa Cruz Biotechnology), mouse monoclonal anti-Flag (Sigma), rabbit polyclonal anti-RIZ1 (Abcam), and rabbit polyclonal anti-β-actin (Sangon Biotech (Shanghai) Co., Ltd.). The bands were quantified by densitometry using ImageJ software.

### Recombinant plasmid construction and luciferase assay

The mouse Akt3 gene promoter region containing CpG island 146 (CpG146) was generated by PCR using mouse genomic DNA. The 5′ primer, bearing a SacI site, was 5′-GCCTCGCCCAGGTGAATGT-3′, and the 3′ primer, bearing a NheI site, was 5′-CGCCAGCAGCGACAGCATCA-3′. The Akt3 promoter fragment was inserted into the pGL3-Basic vector, which includes luciferase as a reporter gene. For the luciferase assay, cells were washed with phosphate buffered saline and harvested with luciferase cell culture lysis reagent. Akt3 promoter activity in the cells was measured with the Luciferase Reporter Assay System using a Sirius luminometer (Titertek-Berthold). Luciferase activity was calculated in relative light units and normalized to the pCMV-RL vector containing the Renilla luciferase as control reporter.

### ChIP assay

We performed ChIP assay using the Agarose ChIP Kit (Thermo Fisher Scientific PierceTM). Cells or mouse liver tissues were cross-linked *in situ* by addition of 16% formaldehyde to a final concentration of 1% and incubated at room temperature for 10 min, and then were incubated with glycine for 5 min. Cells were lysed and digested by Micrococcal Nuclease provided by the kit. The samples were then incubated with anti-Flag, RIZ antiserum 1637^10^, IgG (Thermo Scientific), RNA Polymerase II (Thermo Scientific), H3K9 me1 (abcam), H3K9 me2 (abcam), or H3K9 me3 (abcam) antibody overnight at 4 °C on a rocking platform. Protein A/G plus Agarose were then added to each sample and incubated for 1 hour before washing them with wash buffers. Samples were then treated with Elution buffer, followed by treatment with NaCl and Proteinase K. DNA was then extracted from the digested samples. Extracted DNA sample (the input sample and ChIP DNA sample) was used for PCR amplification using primers specific to promoter fragments of the Akt3 gene and control primers. Positive control primers were from the human GAPDH gene and negative primers were from the Akt3 gene 3′-region. Human and mouse Akt3 core promoter regions were identified using data from Ensemble (http://www.ensembl.org). They were located at human chr1:243848800-243851601 (including CpG island 166) and mouse chr1:177020073-177258203 (including CpG island 146). The primers for qPCR analysis are: human, F1, 5′-CCGTGTGTGGACGAATGC-3′; R1, 5′-AGGTAGGGACCGGAGAGC-3′; product length, 188 bp; F2, 5′-TCAGTGTGTTTGGGTTGG-3′; R2, 5′-AAGGGTGGGGGAAGGAAG-3′; product length, 130 bp; mouse, F1, 5′-GCGAGTCGGTGTTTGGGTT-3′; R1, 5′-CACCTCGCACACGCACACC-3′; product length, 187 bp; F2, 5′-GCCTCGGGTGCCTCGTCC-3′; R2, 5′-TCACCGCCTCAGCTCCGC-3′; product length, 199 bp.

### Expression and Purification of Recombinant Proteins

RIZ1 zinc finger motif fragments were cloned into pGEX-KG vector to express GST fusion proteins pGST-RIZ1-Zf1-3 and pGST-RIZ1-Zf8 containing zinc finger motifs 1-3 and 8, respectively^17^. Plasmids bearing log-phase Escherichia coli cells were induced for 3 h at 30 °C with 0.4 mM isopropyl-1-thio-b-D-galactopyranoside (isopropyl-b-D-thiogalactopyranoside, IPTG). Protein extracts were made and recombinant GST fusion protein was purified with glutathione sepharose 4B (Amersham Biosciences).

### Electrophoretic Mobility Shift Assay (EMSA)

The human and mouse Akt3 promoter probe was synthetic by Sangon Biotech (Shanghai) Co., Ltd. as follows: 5′-TCCGCCCGCAATCTGTGTACCGTGTGTGGACGAATGCTTGCA-3′ (human), 5′-GGGGAGGGGCCGTCTGCGCATGTGGCGGGAGTGCGGGCGA-3′ (mouse). The DNA was included in the products amplified by human and mouse F1 and R1 primers. We used the LightShift^®^ Chemiluminescent EMSA Kit (Thermo Scientific) for the DNA binding analysis. The fragment was end-labeled using biotin 3′ end DNA labeling kit from Beyotime Biotechnology. Then the single strand DNA was annealed with the complementary chain to form a double strand DNA. Biotin end-labeled DNA was incubated with purified recombinant GST fusion proteins, followed by gel electrophoresis on a native polyacrylamide gel and transferred to a nylon membrane (Beyotime Biotechnology). The biotin end-labeled DNA was detected using the Streptavidin-Horseradish Peroxidase Conjugate (Beyotime Biotechnology) and the Chemiluminescent Substrate (Thermo Scientific).

### Transfection experiments

pcDNA3.1/RIZ1-Flag was constructed by our team previously^6^. pcDNA3.1/Zf1-3 and pcDNA3.1/Zf8 were constructed from pGST-RIZ1-Zf1-3 and pGST-RIZ1-Zf8^17^ respectively digested by BamHI and HindIII. The cells transfected with pcDNA3.1 were used as negative controls. The pCMV-RL vector was as an internal standard for the adjustment of transfection efficiency. Including the pGL3-Basic/CpG146, *in vitro* transfections were carried out with Lipofectamine® 2000(Thermo Fisher Scientific) according to the manufacturer’s instruction.

### Statistical analysis

Data were expressed as mean ± standard deviation of the mean (SD). Statistical analyses were performed using SPSS software version 19 (IBM Corporation). Significant differences among the treatment groups were assessed by one-way analysis of variance (ANOVA) and Tukey’s multiple comparison tests. The level *p* < 0.05 were considered to indicate statistically significant differences.
